# Cardiac output limits maximal oxygen consumption, but what limits maximal cardiac output?

**DOI:** 10.1113/EP091594

**Published:** 2025-04-07

**Authors:** Ilkka Heinonen

**Affiliations:** ^1^ Turku PET Centre University of Turku and Turku University Hospital Turku Finland

**Keywords:** blood flow, cardiac output, heart, heart rate, maximal oxygen consumption, myocardium, performance, perfusion, stroke volume

## Abstract

Maximal oxygen uptake/consumption is an important variable determining exercise performance. It is generally considered to be limited largely, but not exclusively, by maximal cardiac output (CO), which limits the ability of heart to pump oxygen‐rich arterial blood to working muscles. Cardiac output is a product of heart rate and stroke volume, which is the amount of blood ejected from the heart by one heart beat. Exercise training, especially of the endurance type, can increase maximal CO substantially. A straightforward way for the heart to increase maximal CO would be to increase maximal heart rate, but this does not happen; instead, maximal heart rate tends to be reduced after training. This is because heart rate is the most important determinant of myocardial oxygen consumption, and ventricular filling and myocardial blood flow (MBF) would be compromised by further increases in heart rate, given that MBF is blunted by contractions and occurs principally during diastole. Myocardial oxygen extraction is already high at rest and is increased further in endurance‐trained athletes, making their hearts even more dependent on increases in MBF. The trained heart therefore also shows reduced MBF, enhanced blood mean transit time and higher myocardial vascular resistance at rest and during submaximal exercise, although MBF reserve is not improved. It follows logically that MBF is an important determinant of myocardial performance, and it is proposed in this review that cardiac afferent sensory nerves might contribute to controlling and limiting heart rate, hence maximal CO, in order to protect the heart from ischaemia.

## Abstract (highlights)

- **What is the topic of this review?**
  This review concerns the factors limiting maximal cardiac output.
- **What advances does it highlight?**
  I discuss myocardial blood flow as a plausible determinant of myocardial performance and propose that maximal heart rate, hence maximal cardiac output, might be controlled and limited by myocardial sensory afferent nerves, which prevent the heart from experiencing ischaemia.

## 1 INTRODUCTION

Maximal oxygen consumption (${\dot{V}}_{O_{2}\max}$) is an important (albeit not the only) factor limiting maximal aerobic fitness, performance and even survival in health and disease (Heinonen, Kalliokoski et al., 2014; Joyner & Casey, 2015; Joyner & Dominelli, 2021; Koga et al., 2014; Levine, 2008; Lundby et al., 2017). Maximal oxygen consumption describes the ability of the lungs to take oxygen from air to be saturated into arterial blood, the ability of the heart and vasculature to deliver it to working muscles and, ultimately, the ability of muscle mitochondria to take and consume it to provide energy for working muscles, because muscle work cannot be sustained for long without oxygen. The limiting factors might lie at all possible sites of this chain (Peters et al., 2023; Poole & Musch, 2023; Poole et al., 2022; Wagner, 1996, 2000b, 2023) and can be affected by age, sex, training status, environmental conditions (such as altitude or air temperature) and disease state, but it is widely considered and accepted that an important factor limiting ${\dot{V}}_{O_{2}\max}$ is maximal cardiac output (CO), the ability of the heart to pump blood and thus oxygen to maximally working skeletal muscles (Heinonen, Kalliokoski et al., 2014; Joyner & Casey, 2015; Joyner & Dominelli, 2021; Koga et al., 2014; Levine, 2008; Lundby et al., 2017). The heart cannot pump more blood forwards than it receives back from veins; therefore, ${\dot{V}}_{O_{2}\max}$ is usually obtained in an exercise that is heavy in intensity but is in nature dynamic and rhythmic rather than static or where force‐production times are long. This maximizes venous return to the heart by muscle pump mechanisms when the heart is also maximally stretched, making it pump and empty more forcefully and completely. When >50% of skeletal muscles are engaged in exercise, the capacity of muscle mitochondria far exceeds the ability of the heart to supply them with oxygen (Boushel & Saltin, 2013), making CO the most important factor limiting maximal aerobic fitness in large‐muscle‐mass exercise, especially in trained people (Broxterman et al., 2024). It is widely acknowledged that limiting factors for ${\dot{V}}_{O_{2}\max}$ can also exist at the periphery, and readers are encouraged to delve into the excellent reviews about diffusion limitations in the lungs and skeletal muscle for historical and other perspectives (Peters et al., 2023; Poole & Musch, 2022, 2023; Poole et al., 2022; Wagner, 1996, 2000a, 2000b, 2023). Nonetheless, given that CO also obviously reaches its individual limits during heavy exercise, this review focuses on that and tries to propose and speculate on plausible explanations why CO does not increase infinitely.

## 2 FACTORS LIMITING MAXIMAL CARDIAC OUTPUT

Cardiac output is the product of heart rate and stroke volume, which is the amount of blood that is released into the arterial circulation from the heart by one heart beat. Maximal heart rate is typically ∼200 beats/min in young adult humans, although ageing, for instance, reduces it. It is apparent that genetics also contribute to the size of the heart and thus its performance, but what are the training adaptations that allow highly trained endurance athletes to reach as high as 30–40 L for maximal CO that distinguish them from normal untrained persons, whose maximal CO is some 15–25 L/min? One could assume that a straightforward mechanism and training adaptation would be to increase maximal heart rate to 250, 300 or 400 beats/min, but this does not happen. If anything, maximal heart rate is reduced in response to endurance training (Whyte et al., 2008; Zavorsky, 2000) that improves performance markedly. This is likely to be for two important reasons: (1) the heart beat blunts myocardial perfusion, which happens almost solely during diastole; and (2) heart rate is the most important determinant of myocardial metabolic and thus oxygen demand, and normal function of the heart relies almost solely on aerobic energy production.

**FIGURE 1:**
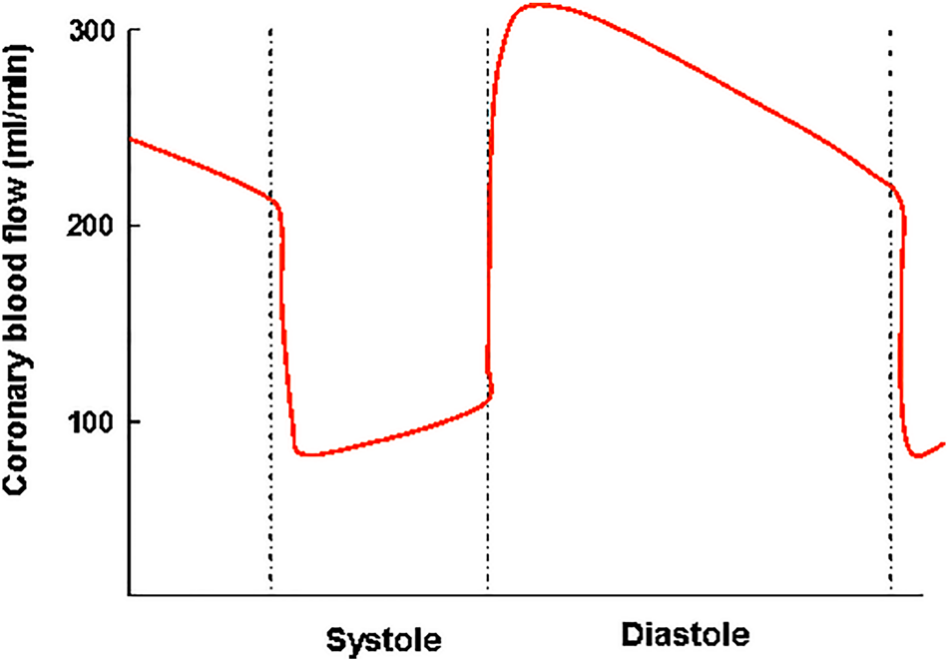
The effect of cardiac muscle contraction‐shortening on coronary blood flow (modified from Guyton & Hall, 2000: p. 227 and published earlier in the PhD thesis of Heinonen, 2010). During systole, cardiac muscle contraction impairs blood flow in the arteries supplying the myocardium, and there is flow only in the epicardial layer of the heart. During diastole, compression is released, and all the layers of the left ventricle are supplied by blood rich in oxygen. A similar pattern of flow occurs in skeletal muscle during the contraction–relaxation cycle.

Similar to a single contraction of skeletal muscle, blood flow is almost completely blocked during systole in the heart and myocardial perfusion happens largely during diastole, (i.e., the relaxation phase of the heart; Figure 1). It follows that as heart rate increases, the heart spends more and more time during the contraction phase, which blunts its oxygen supply. Furthermore, with increasing heart rate the time spent in diastole, allowing myocardial perfusion, shortens. Given that increasing the heart rate also increases the metabolic and oxygen demands enormously, it follows logically that it is not wise for the myocardium to increase its performance by increasing (maximal) heart rate, because this would drastically compromise its own performance. Therefore, the main adaptation of the myocardium is to increase its size and maximal stroke volume. Myocardial hypertrophy occurs to some extent as increased wall thickness, but largely by eccentric hypertrophy, which is evident by the increased length and diameter of both left and right ventricles. This leads to a condition in which a large stroke volume is already evident at rest, while resting and submaximal heart rates are reduced, leading to largely unchanged CO at rest and during submaximal exercise, but superior stroke volume and thus CO during maximal exercise. As a result, the most distinct feature of highly trained and performing endurance athletes is a large and compliant heart that relaxes quickly (Levine, 2008), which therefore pumps blood into arteries with maximal volumes and simultaneously allows more time for its own filling and also time for myocardial oxygen supply by also limiting its own oxygen demand (reduced maximal heart rate).

Thus, interplay between blood pumping capacity of the heart also takes into account its own filling and perfusion, making myocardial oxygen demand and supply crucial factors to consider both for the function of the heart and for whole‐body performance. This interplay is likely to be species dependent; for instance, in rodents the heart rate can be 500–600 beats/min, with no signs of ischaemia. However, their cardiac walls are also much thinner and their myocardial vasculature is different from that of humans, meaning that maximal heart rate numbers cannot be compared directly. Furthermore, it is generally considered that the heart of pigs might resemble the human heart the closest, whereas horses and dogs are very aerobic species that have a large increase in arterial oxygen content during maximal exercise (Poole & Erickson, 2011), which also makes them distinct from humans, especially with regard to their extraordinary pumping capacities and very fast maximal heart rates. As reviewed comprehensively by Duncker and Bache (2008), (large) animal studies have elucidated that exercise training alters the regulation of coronary resistance vessel vasculature, which might contribute to improving blood flow during diastole such that better matching of blood flow to demand occurs and improves the performance of the myocardium with training even if maximal coronary reserve is not altered. These alterations include structural changes in arterioles, neurohumoral control (increased α‐adrenergic coronary tone, but unaltered β‐adrenergic and parasympathetic tone), augmented myogenic tone and endothelium‐dependent vasodilatation (largely nitric oxide) and decreased extravascular compressive forces, which lead to reduced resting and submaximal exercise blood flow (Duncker & Bache, 2008). Nevertheless, human studies have also been performed to elucidate the (endurance) exercise training adaptations in myocardial oxygen supply and their regulation.

## 3 MYOCARDIAL BLOOD FLOW AT REST AND DURING EXERCISE IN THE ENDURANCE‐TRAINED HUMAN HEART

**FIGURE 2:**
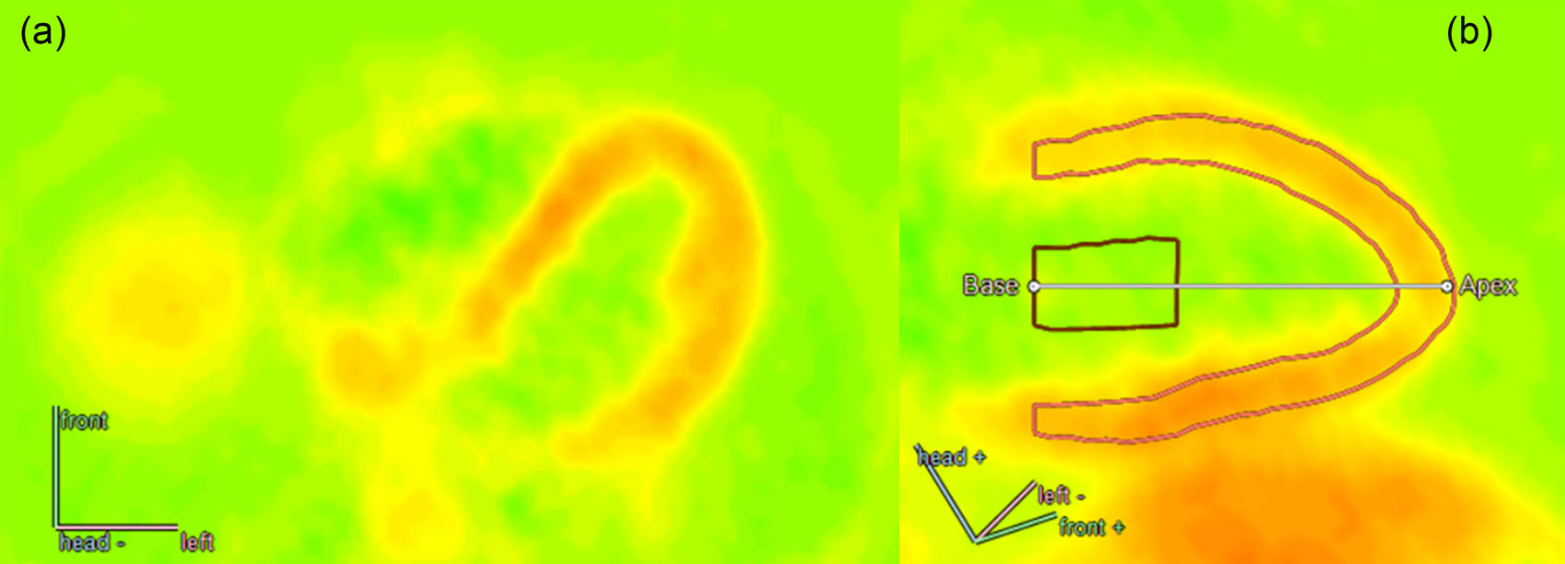
(a) Cross‐sectional positron emission tomography (PET) blood flow image of thoracic region, where the left and right ventricles are visible, as analysed by Carimas software. (b) Region of interest determination by Carimas for measurement of blood flow in the left ventricular myocardium. PET cannot measure beat‐by‐beat fluctuations of coronary blood flow but is considered the gold standard for the measurement of myocardial blood flow in humans (Camici & Crea, 2007). In addition to being able to measure only average blood flow over several minutes, another major limitation of PET is that myocardial blood flow cannot be measured during upright exercise, which is the most typical posture in which most exercise and exercise training occurs.

Given that oxygen extraction is already very high (fractional O_2_ extraction of 60%–80%) even in the resting (albeit continuously beating) heart, this means that almost all the increase in myocardial oxygen supply must be met by increasing myocardial blood flow (MBF) (Duncker & Bache, 2008). A tight correlation between heart rate and MBF is evident in health and disease, such as in breast cancer patients and healthy control subjects (Koivula et al., 2024). The MBF (Figure 2) is already very high at resting heart rates, ∼1 mL/min/g, thus many‐fold higher than in skeletal muscle, and it can increase 3‐ to 5‐fold in response to maximal exercise. Although a large stroke volume is the hallmark adaptation in response to endurance training, we have shown that like the training adaptation in skeletal muscles, highly trained endurance athletes show enhanced myocardial oxygen extraction both at rest and during submaximal exercise, without changes in haemoglobin 2,3‐diphosphoglycerate (Heinonen, Kudomi et al., 2014). This means that they are even more heavily reliant on the increase in MBF to meet the metabolic demands during maximal exercise. In addition to higher oxygen extraction fraction (OEF), highly trained endurance athletes also show enhanced blood mean transit time in myocardial capillaries (Heinonen, Kudomi et al., 2014), thus allowing more time to release oxygen when blood is transported through the myocardium. The MBF is lower both at rest and during submaximal exercise, but myocardial blood volume, a proxy for myocardial capillary or vascular density/volume in humans, is not different from that of healthy untrained control subjects (Heinonen, Kudomi et al., 2014). This is in line with animal data suggesting that not capillary density but three‐dimensional arterial vascular structure and gas diffusion capacity are enhanced in the trained state (Laughlin et al., 2012), whereas maximal MBF is neither reduced or supranormal but is similar to that of untrained persons (Heinonen et al., 2008). However, we have also seen some indications that, similar to diseased states involving pathological myocardial hypertrophy, higher left ventricular mass appears to influence MBF reserve, even when MBF is measured per gram of myocardium (Laaksonen et al., 2014). The efficiency of left ventricular (LV) work is not, however, changed by training (Heinonen, Kudomi et al., 2014). Furthermore, the proportions of LV blood flow and oxygen consumption from whole‐body CO (total LV blood flow divided by CO) and oxygen consumption (LV oxygen consumption divided by whole‐body oxygen consumption), respectively, are not changed by training, although both proportion variables are reduced from rest to exercise (Heinonen, Kudomi et al., 2014). Interestingly, total LV oxygen consumption per heart beat is not affected by training in humans, but it increases from rest to low‐ to moderate‐intensity exercise (Heinonen, Kudomi et al., 2014), probably owing to increased afterload and/or contractility.

It is often considered to be an improvement if exercise training increases resting or submaximal limb blood flow, especially in diseased populations. However, this is not a typical training response after endurance training, which leads to reduced blood flow, especially during submaximal exercise (Delp, 1998; Proctor et al., 2001). If blood flow is too high or fast, haemoglobin does not release its oxygen optimally, although there is no indication that oxygen extraction would be compromised even during very high (skeletal muscle) blood flows (Richardson et al., 1993). Another important characteristic in human myocardium is that myocardial vascular resistance is higher in endurance athletes, both at rest and during submaximal exercise (Heinonen, Kudomi et al., 2014). My interpretation is that this is to prevent the myocardium from ‘overperfusion or luxurious’ MBF in relationship to its metabolic demands. This is comparable to conduit arteries after endurance training. Furthermore, we (Heinonen, Kudomi et al., 2014) and others (Iellamo et al., 2002; Neri Serneri et al., 2001) have found evidence that sympathetic tone is also increased in highly trained endurance athletes, which most probably acts to constrict epicardial arteries at rest. In a seminal study, Haskell et al. (1993) showed that ultra‐endurance runners had larger epicardial arteries during nitroglycerin‐induced vasodilatation, but not at resting baseline. Similar coronary artery diameters at rest are most likely to be an adaptation to prevent the heart from overperfusion, and the capacity will be used only during maximal exercise. Control persons in this study were patients referred for coronary angiography, but who were free of obstructive coronary artery disease. Nowadays, we know that microvascular dysfunction is also a common feature of coronary artery disease (Sorop et al., 2017) and that plaques can develop in smaller arteries before they are observed in large epicardial arteries (Sorop et al., 2016). Left ventricular mass was somewhat higher (but this was not statistically significant) in athletes, but unfortunately the authors did not normalize dilated epicardial coronary arterial sizes to LV mass. In an exercise intervention study, it was later shown that coronary artery size increases with increases in LV mass, although it also appeared that the increase in the coronary artery size did not keep up with the increase in LV mass (Windecker et al., 2002) when LV mass values started to reach the LV mass values that are observed in highly trained and performing endurance athletes whose MBF we have measured for MBF reserve (Heinonen et al., 2008). In studies by Haskell et al. (1993) and by Windecker et al. (2002), in which the trained population was lower in aerobic performance calibre (and older), vasodilated LV coronary artery size was correlated with maximal aerobic fitness (in Haskell et al. (1993) study in runners), which is in line with that observed in skeletal muscle vasculature (Heinonen et al., 2010).

## 4 MYOCARDIAL BLOOD FLOW AND ISCHAEMIA‐PROTECTION MECHANISMS AS LIMITING FACTORS?

As mentioned earlier, myocardial oxygen demand is determined mainly by heart rate, but also by afterload (thus, systolic blood pressure when estimated non‐invasively) and myocardial contractility. Contractility is very hard to measure, especially in humans, and it may or may not be increased by training, but it is considered to be slightly enhanced towards maximal exercise. Given that heart rate and systolic blood pressure also increase markedly towards maximal exercise, and myocardial oxygen demand must be met almost solely by MBF, it follows logically that MBF is one of the most (if not the most) crucial factors determining myocardial performance.

Given that MBF reserve is not increased in highly trained and performing endurance athletes (Heinonen et al., 2008), the heart must have certain ‘protection’ mechanisms in order that hypoxia and, especially, ischaemia do not generally happen during maximal exercise in healthy humans. This does not necessarily mean or suggest the existence of a central governor, but suggests that as ischaemia‐type symptoms are very rare, especially in highly performing endurance athletes, some sensory information must exist that controls the heart and prevents it from experiencing an inadequate oxygen supply (Noakes, 2000). Although the role of skeletal muscle afferents has been studied and reviewed extensively (Fisher, 2014; Teixeira & Vianna, 2022; Teixeira et al., 2020), to the best of my knowledge the role of myocardial afferent nerves has not been investigated as sensors for myocardial and whole‐body performance, which could communicate metabolic, mechanical and oxygen levels and limit cardiac work before ischaemia develops.

**FIGURE 3:**
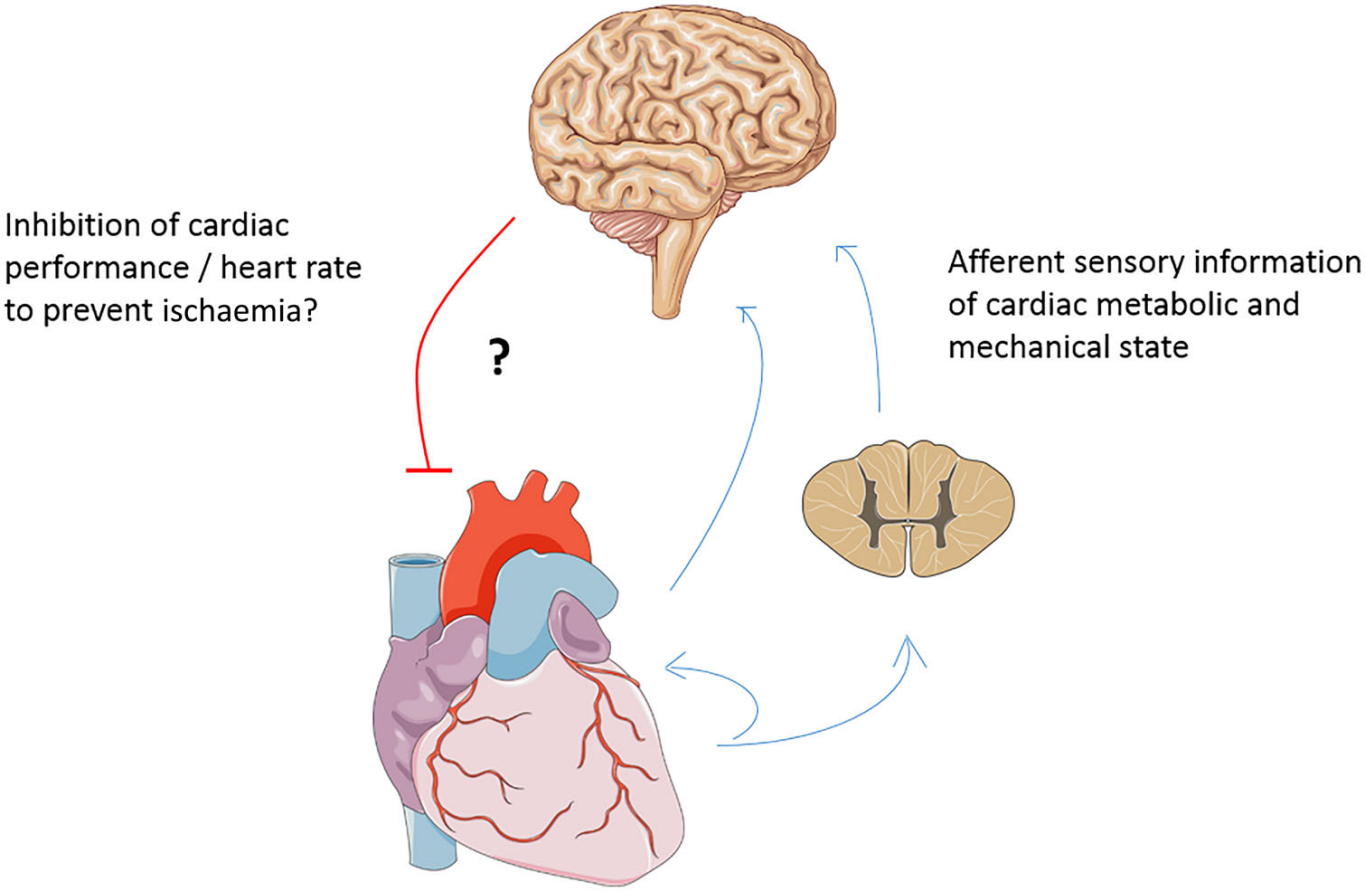
Schematic diagram of myocardial afferent sensory nerves controlling heart so as not to exceed its limits and to prevent the development of ischaemia and other severe perturbations during maximal exercise, causing a ‘ceiling effect’ for maximal heart rate.

Cardiac afferent nerves are sensory nerves that convey heart information: (1) within the myocardium or (2) directly or (3) via the spinal cord to the brain (Armour, 2004, 2008; Clyburn et al., 2022; Duraes Campos et al., 2018; Fedele & Brand, 2020; Fukuda et al., 2015; Kimura et al., 2012; Malliani et al., 1981; Moore et al., 2022; Paintal, 1973; Spyer, 2002; 1979; Van Weperen & Vaseghi, 2023; Vance & Bowker, 1983; Zandstra et al., 2021). Stimulation of vagal afferent nerves leads to sympathoinhibitory, cardioinhibitory and vasodepressor responses. The heart also has sympathetic afferent nerves, which are excitatory, but their role during exercise has not been characterized. Counterintuitively, particularly during myocardial ischaemia, but also at maximal heart rate, activation of cardiac sympathetic afferent nerves would lead to cardioaccelelator and vasoconstrictor responses, which could cause further mismatch between oxygen supply and demand. Coronary arteries are also innervated by afferent nerves, but apparently chemoreceptors are located only or almost only in the aortic and carotid bodies and not in the coronary arteries, although it is obvious that myocardial afferents can also sense O_2_ pressure in the interstitial space when it is reduced owing to increased metabolic demand. However, as pointed out in the review by Shepherd (1985), there are chemoreceptors in the myocardium that have a direct arterial supply from coronary arteries, which thus directly ‘see’ arterial oxygenation in close proximity to the myocardium. Nevertheless, metabolic disturbance or danger of it might not be the only stimulus for myocardial afferents, because they are also sensitive to changes in the cavity pressures and other mechanical stimulus. Afferents are also found at the venous–atrial junctions (interface connecting the veins and atria) in addition to the chamber walls (Shepherd, 1985). It is also likely that in the healthy heart and body these afferent sensors are so sensitive and fine‐tuned as to detect even subtle changes in metabolic and mechanical states that they will not allow the heart to overreach and ischaemia to develop during maximal exercise (Figure 3).

Although challenging, this area should be investigated to refute or support the idea of myocardial afferent communication as a limiting factor for maximal CO. An indication of this afferent or sensory information could be that although coronary artery blood flow increases drastically in response to systemic hypoxia at rest (Heinonen, Luotolahti et al., 2014), maximal heart rate is reduced in hypoxia owing to increased parasympathetic activity (Boushel et al., 2001), which is likely to act to limit cardiac metabolism (maximal heart rate) in the face of hypoxic arterial blood such that maximal MBF capacity is not exceeded and ischaemia does not develop. These afferent nerves might produce a ‘ceiling effect’ for further increase in maximal heart rate at the risk of MBF and oxygen consumption mismatch and could potentially explain why maximal heart rate is not increased but is usually lowered by training and why CO capacity is improved by increase in stroke volume. It might be that no direct vagal activation is triggered at maximal heart rate, but afferent vagal sensors could limit further increases in sympathetic activation. Testing of this hypothesis would require direct measurements of the firing of these afferent nerves in non‐human species, because, for instance, non‐invasive heart rate variability measures are questionable for measuring cardiac autonomic actions (Monfredi et al., 2014), let alone these afferent inputs. Sympathetic afferent nerves are stimulated principally by myocardial ischaemia, which does not usually develop in the healthy heart, suggesting that it might indeed be vagal afferents that are being activated when the heart is facing its limits.

It must be emphasized that it is generally considered, based on detailed animal studies, that coronary blood flow reserve is substantial, especially in the healthy heart (Barnard et al., 1980, 1977). This is also the case in humans, as reviewed. However, to the best of my knowledge no studies have been performed in humans to measure MBF during maximal exercise and cessation of that exercise with direct sampling of oxygen partial pressures in arterial blood and in the coronary sinus. Only these measurements could tell us directly about the dynamics of myocardial oxygen supply and utilization and whether the heart is completely prevented from hypoxia during maximal exercise. For this reason, I have chosen to use the word ‘ischaemia’ rather than ‘hypoxia’, because it cannot currently be ruled out that not even benign hypoxia (lowered oxygen supply, blood flow × arterial oxygen content, in comparison to pre‐fatigue conditions) could develop in the myocardium during fatiguing short‐term exercise, in which a decrease in arterial oxygen saturation is also not uncommon, especially in highly trained endurance athletes (Dempsey & Wagner, 1999; Peters et al., 2023). Furthermore, it might be that the cardiac afferent nerves that were highlighted here are not the only afferent inputs that contribute to limit maximal heart rate and therefore maximal CO and whole‐body performance. It is plausible that the body integrates all afferent sensory information, stemming also from working skeletal muscles and elsewhere (thermal effects), and adjusts its capacity accordingly to prevent risks for health, such as myocardial ischaemia.

In addition to the LV, the right ventricle (RV) is at least as important as the LV for exercise performance (La et al., 2017; Naeije & Chesler, 2012). We have measured MBF, OEF and myocardial oxygen consumption in the RV at rest and during submaximal exercise in healthy young men (Kudomi et al., 2019) and shown, among other things, that OEF in the RV increases in response to acute exercise, but the effects of exercise training on the RV, other than increased RV size, remain to be characterized. From the RV, blood travels to the lungs. Although pulmonary blood flow reserve appears not be increased in highly trained endurance athletes, their pulmonary blood flow is somewhat more homogeneous (Heinonen, Savolainen et al., 2013), possibly reflecting higher capillary density in the endurance‐trained lungs. However, I am not aware of any studies, either cross‐sectional or interventional, that have addressed the effect of (endurance) training on pulmonary vasculature in humans to confirm the speculation about the increased capillarity in the trained lung. It is generally believed in the field that exercise training does not increase lung size, except, perhaps, in swimmers throughout puberty, although not all studies support even that view (Bovard et al., 2018). Therefore, the training‐induced increase of pulmonary O_2_ uptake is most probably attributable to increased functional pulmonary capillary volume owing to greater CO, and therefore pulmonary vascular pressures distending extant capillaries and increasing capillary haemoglobin content.

## 5 HOW TO TRAIN TO IMPROVE MAXIMAL STROKE VOLUME, HENCE MAXIMAL AEROBIC FITNESS

Given that blood doping is, naturally, forbidden, athletes and other exercisers could try to improve their oxygen carrying and supply capacity by optimal and progressive endurance training and, especially, altitude training (Levine & Stray‐Gundersen, 2006). Given that increasing haemoglobin mass naturally in absolute terms is modest at best, larger improvements in oxygen supply capacity can be obtained by increasing maximal stroke volume. If CO and heart rate are not measured accurately, stroke volume is challenging to measure reliably by imaging methods, such as ultrasound, and it is also affected by sex, age and training status, and different methods to measure it add to the complexity, hence no firm conclusions can be said how it behaves in response to incremental exercise until exhaustion (Vella & Robergs, 2005). In general, it appears to be the case and is commonly believed that in younger persons, females and persons with lower fitness and/or training status, stoke volume increases only modestly or does not increase at all in response to incremental exercise, whereas in fitter and more trained persons it increases close to or until maximal exhaustive exercise (Vella & Robergs, 2005). Furthermore, given that stretching of the myocardium by blood is the hallmark stimulus for the improvement of stroke volume, one could suggest that the best stimulus to train stroke volume is to exercise at those exercise intensities at which stroke volume is maximal or close to maximal. In less fit people, this would mean lower exercise intensities, at which volume overloading is also easier to maintain, but when fitness increases, higher exercise intensities are usually required to increase stroke volume further. Naturally, wise endurance athletes do not train only at low, moderate or high exercise intensities, but they combine all these different exercise intensities to improve their fitness and performance. For the development of stroke volume, venous return should be maximized, meaning that exercise should be rhythmic and dynamic in nature rather than static or slow movements with high force requirements.

Other major training adaptations of endurance training in the periphery are increased capillary density and mitochondrial capacity in skeletal muscles (Hellsten & Nyberg, 2015). This makes it easier for blood and haemoglobin to travel close to working skeletal muscle fibres and release oxygen. Thus, increased OEF is also a typical endurance training adaptation (Kalliokoski et al., 2001). Interestingly, this is the case in adults but not in children, where improvements in maximal aerobic fitness are attributed solely to increased stroke volume, which is evident as much lower submaximal exercise heart rates after training intervention (Mandigout et al., 2001, 2002). The ${\dot{V}}_{O_{2}\max}$ is also important in children, especially in terms of health, but maturation and a reasonable amount of versatile physical activity appear to be more important for it than hard exercise training (Heinonen, 2024). In adults, however, there could also be an improvement in matching of blood supply to the metabolic demand of muscles, contributing to improved OEF, but direct evidence for this is largely lacking (Heinonen et al., 2015; Koga et al., 2014). Skeletal muscles are often considered to be a ‘sleeping giant’ (Joyner, 2004), meaning that their capacity to receive blood far exceeds the capacity of the heart to supply it, and therefore skeletal muscle vasculature is always constrained by the sympathetic nervous system in order that that blood pressure does not drop during heavy exercise. However, when we administered phentolamine (an α‐adrenergic receptor inhibitor blocking sympathetic nervous activation) into the femoral artery before and during exercise to study the sympathetic nervous constraints of exercise hyperaemia, blood flow was increased only in inactive skeletal muscles and not in the exercising muscles (Heinonen, Wendelin‐Saarenhovi et al., 2013), suggesting that nervous constraint occurs in inactive rather than active musculature. However, this was the case during small‐muscle‐mass, fairly moderate‐intensity exercise, and this phenomenon remains to be studied during high‐intensity, whole‐body exercise. Nevertheless, when Calbet et al. (2006) infused the most potent vasodilator known, ATP, into the femoral artery during maximal exercise, ${\dot{V}}_{O_{2}\max}$ did not improve. In fact the OEF was reduced, meaning that vasodilatation occurred in inactive muscle fibres and it disturbed the precise matching of blood supply to metabolic demand, which is of crucial importance during maximal exercise. Thus, simply increasing blood flow by pharmacological or other means does not necessarily lead to improved exercise performance, because the phenomenon is complex.

When exercise is not maximal and the duration of exercise increases, peripheral local muscular adaptations become more important than the ability of the heart to pump blood to muscles. Therefore, for longer‐duration races and training sessions it is also important to train efficiency and movement economy and lactate threshold by sport‐ and event‐specific training speeds. Critical speed and oxygen uptake kinetics should not be forgotten either, and their relevance in contributing to exercise capacity is reviewed in detail elsewhere (Poole et al., 2021). Finally, and in comparison to the heart, limbs are not as crucial in terms of oxygen and can easily be driven close to the ischaemic state, as experienced by any exerciser when effort becomes supramaximal for too extensive a period by increased speed, power or strength levels. This also highlights the central role of the heart and, possibly, afferent sensory nerves protecting its limits during maximal exercise.

## 6 CONCLUSIONS

In conclusion, given that heart rate is the most important determinant of myocardial oxygen consumption and ventricular filling, MBF would be compromised with further increases in (maximal) heart rate because MBF is blunted by contractions and occurs during diastole. Therefore, the hallmark adaptation of the heart to endurance exercise training, which also results in improved fitness, is an increase in stroke volume that results in enhanced CO, while maximal heart rate is maintained or reduced. Furthermore, myocardial oxygen extraction is already high in the resting state and is increased further in endurance‐trained athletes, making their hearts even more dependent on increases in MBF. The trained heart therefore also shows a reduced MBF and an enhanced blood mean transit time and myocardial vascular resistance at rest and during submaximal exercise, while MBF reserve is not improved. It is proposed here that the heart faces a ceiling effect at maximal exercise such that myocardial oxygen consumption does not to exceed its functional limits, and it prevents the development of ischaemia. It is also proposed that maximal CO is more limited structurally rather than functionally and that interplay and matching exist to allow CO to reach its maximum without allowing ischaemia to develop. It is proposed that this is regulated by afferent sensory mechanisms that tightly match MBF to myocardial oxygen demand (Figure 3). Therefore, the main adaptation in response to endurance‐type exercise training is a lower (not higher) maximal heart rate.

## AUTHOR CONTRIBUTIONS

Sole author.

## CONFLICT OF INTEREST

None declared.
